# Prediction of Aortic Stenosis Progression by ^18^F-FDG and ^18^F-NaF PET/CT in Different Aortic Valve Phenotypes

**DOI:** 10.3389/fphar.2022.909975

**Published:** 2022-05-24

**Authors:** Patimat Murtazalieva, Darya Ryzhkova, Eduard Malev, Ekaterina Zhiduleva, Olga Moiseeva

**Affiliations:** ^1^ Non-coronary Heart Disease Department, Almazov National Medical Research Centre, Saint Petersburg, Russia; ^2^ Department of Nuclear Medicine and Theranostics, Almazov National Medical Research Centre, Saint Petersburg, Russia

**Keywords:** aortic stenosis, bicuspid aortic valve, calcification, aortic stenosis progression, positron emission tomography, 18 F -sodium fluoride, 18 F -fluorodeoxyglucose

## Abstract

**Background:** Different imaging techniques, such as echocardiography (ECHO) and CT, allow to assess aortic stenosis (AS) severity and could be used to study its progression. But only PET/CT open opportunities to assess activity of valvular inflammation and calcification *in vivo*. The aim of this study was to assess prognostic value of valvular inflammation and calcification measured by ^18^F-FDG and ^18^F-NaF PET/CT in patients with tricuspid (TAV) and bicuspid aortic valve (BAV).

**Methods:** The study included 71 patients aged 40–70 years with mild, moderate and severe asymptomatic calcific AS. Patients were divided into two groups according to valve morphology: with BAV and TAV. All patients underwent standard ECHO, CT calcium scoring PET/CT with ^18^F-NaF and ^18^F-FDG. All patients were evaluated during a follow-up visit with evaluation of ECHO parameters. (16.8 ± 4.2 months).

**Results:** TAV and BAV groups were comparable in AS severity by ECHO (peak aortic jet velocity (Vmax): 2.90 [2.60; 3.50] vs. 2.96 [2.55; 3.31] m/s, *p* = 0.83). TBR max ^18^F-FDG did not vary in TAV and BAV patients (1.15 [1.06; 1.23] vs. 1.11 [1.03; 1.20], *p* = 0.39). Both groups did not differ in valvular calcification degree (Agatston score 1,058 [440; 1798] vs. 1,128 [533; 2,360], *p* = 0.55) and calcification activity assessed by ^18^F-NaF uptake level (TBR max 1.50 [1.30; 1.78] vs. 1.48 [1.27; 1.83], *p* = 0.97). ^18^F-NaF TBR max was associated with AS severity measured by Vmax in men and women with TAV (r = 0.54; *p* = 0.04 vs. r = 0.53; *p* = 0.03). In BAV group this relationship was true only in female patients (r = 0.1; *p* = 0.67 vs. r = 0.7; *p* = 0.0004). There was no association between Vmax and TBR max ^18^F-FDG was revealed in TAV and BAV groups. During follow-up period, the most important positive predictors of AS progression in TAV obtained by multinomial logistic regression analysis were Vmax, and ^18^F-NaF TBR. Whereas in BAV the highest predictive value showed model included age and Vmax.

**Conclusion:** ^18^F-NaF PET/CT may be considered as the valuable predictor for hemodynamic progression of calcific AS in case of TAV. ^18^F-FDG PET/CT does not play a significant role to predict the AS progression.

## Introduction

Aortic stenosis (AS) represents the most common indication for valve surgery as well as for catheter interventions in valvular heart disease (Iung et al., 2019). Despite many recent advances in AS management, no pharmacotherapy has demonstrated its efficacy to prevent AS progression (Pawade et al., 2021; Vahanian et al., 2022). The incidence of AS has been increasing, that may contribute to significant burden on healthcare system.

The most common etiology of AS is known to be a calcification of the tricuspid aortic valve (TAV) or congenital heart defect - bicuspid aortic valve (BAV). Intraoperatively, BAV is represented in 50% of AS surgical interventions, while TAV is observed in 30–40% (Roberts and Ko, 2005). Other etiological factors, such as chronic rheumatic heart disease, infective endocarditis and single-leaf aortic valve are less common.

The specific factors that may facilitate aortic valve calcification remain underinvestigated whereas the underlying pathogenetic mechanisms have not been well defined, so treatment options to prevent AS progression are lacking. It is known that conventional risk factors for the atherosclerosis such as age, sex, hypertension, elevated serum cholesterol, smoking and diabetes are also associated with aortic valve calcification (Stewart et al., 1997; Eveborn et al., 2014; Martinsson et al., 2014; Yan et al., 2017). Notably, early initiation and progression of AS in those with TAV correlate mainly with cardiometabolic risk factors, whereas in BAV subjects, the genetic predispositions and abnormal valve morphology have an independent contribution to the AS progression (Shen et al., 2020).

Recent histology and molecular biology studies support the hypothesis for the aortic valve calcification as a part of the immuno-inflammatory process associated with endothelial cell damage, accumulation of oxidized low-density lipoproteins and inflammatory signaling pathway activation (Venardos et al., 2014; Zheng et al., 2019). At the next stage, the progression of AS contributes to the transition of valvular interstitial cells to an osteoblast-like phenotype with the subsequent aortic valve calcification (Bogdanova et al., 2018). Although the pathogenesis of AS goes far beyond just hemodynamic valve damage, this mechanism plays a significant role in patients with BAV.

Despite the fact that the pathophysiological mechanisms of calcification are being actively studied, there is still no effective preventive tactics to avoid surgical intervention. Further experimental researches, both *in vitro* and *in vivo*, as well as clinical studies are required for adequate treatment and prophylaxis of valvular calcification. Contemporary diagnostic tools including positron emission tomography (PET) and PET/computed tomography (CT) represent new opportunities to evaluate the inflammation and calcification process in real clinical settings.

Being integrated molecular anatomic imaging modality PET/CT technique could provide detailed information about the specific disease activity *in vivo*. In recent years, PET has utilized two radioactive tracers: ^18^F-fluorodeoxyglucose (^18^F-FDG) to estimate the degree of inflammation of the aortic valve in patients with AS and ^18^F-fluoride (^18^F-NaF) to assess calcification. ^18^F-FDG is a glucose analogue which is accumulated in metabolically active cells and represents a sensitive marker of vascular inflammation (Tawakol et al., 2006; Honda et al., 2016). ^18^F-NaF is a bone tracer that binds to hydroxyapatite, a crucial component of valvular calcification with greater surface area in regions of microscopic calcification. Higher valvular ^18^F-NaF uptake is independently associated with more rapid disease progression and therefore represents a potential biomarker of AS disease activity (Dweck et al., 2014). ^18^F-NaF PET/CT can also be considered as diagnostic tool providing assessment of calcification level along with estimation of treatment efficacy. However, there no data about valve inflammation and calcification activity *in vivo* measured by PET/CT using outlined above radiotracers in BAV and TAV patients and their prognostic value.

The aim of this study was to assess prognostic value of valvular inflammation and calcification measured by ^18^F-FDG and ^18^F-NaF PET/CT in patients with TAV and BAV.

## Materials and Methods

### Patient Population

Study enrolled 71 patients aged 40–70 years with mild, moderate and severe asymptomatic calcific AS. Patients with rheumatic heart disease, infective endocarditis, severe chronic kidney disease, prior thoracic radiotherapy or aortic valve interventions were not included. Patient enrollment was performed in 2015–2016. The study protocol was approved by the research ethics committee of the Almazov National Medical Research Centre, and all participants provided written informed consent.

As part of the baseline examination, information on conventional cardiovascular risk factors including age, sex, hypertension, diabetes, lipid profile, smoking, family history of cardiovascular diseases was analyzed. Standard echocardiography using the Vivid 7.0 system (GE, United States) was performed to all participants by the same team of sonographers and cardiologists, while all images were analyzed by experienced readers at the same laboratory according to the American Society of Echocardiography Guidelines (Baumgartner et al., 2009). Doppler echocardiographic values of AS severity included peak aortic jet velocity, mean gradient obtained by the Bernoulli formula, and aortic valve area calculated by the continuity equation and indexed to body surface area (iAVA). Left ventricular systolic function was assessed using left ventricular ejection fraction as measured by the biplane Simpson method. Stroke volume was also calculated and indexed to body surface area.

### Multimodal Imaging Using Combined ^18^F-FDG and ^18^F-NaF PET/CT

PET/CT with ^18^F-FDG and ^18^F-NaF was performed in all patients (Discovery 710, GE). The study protocol of ^18^F-NaF PET/CT included intravenous injection of a 300 MBq ^18^F-NaF during at least 0,5–1 min. CT scan for attenuation correction was performed in 90 min after radiotracer injection, during that period the patient was positioned on the bed in the supine position, and the patient’s heart was located in the center of the field of view. Immediately after the CT series РЕТ emission scanning was performed in static mode without changing of the patient’s position. PET-CT with ^18^F-FDG required special preprocedural planning. To avoid the physiological ^18^F-FDG uptake in myocardium the patient should keep to low-carbohydrate diet within 3 days before the PET procedure. The study was performed after 12 h of fasting. The study protocol included intravenous injection of a 300–370 MBq ^18^F-FDG during at least 0,5–1 min. The CT scan for attenuation correction was performed in 60 min after radiotracer injection, during that period the patient was positioned on the bed in the supine position, and the patient’s heart was located in the center of the field of view. Immediately after the CT series PET emission scanning of the same area was performed in static mode without changing the patient’s position. PET-CT images were reconstructed on the 128 × 128 matrix in the 300 mm field using the iterative reconstruction algorithm.

Semiquantitative analysis of the PET/CT results was implemented. Image interpretation was performed by two independent experts (experienced in cardiovascular imaging). The circular region of interest (ROI) of 3.52 square millimeters was drawn around areas of maximal radiotracers uptake in the valve (target) and then maximal and mean levels of standardized uptake value (SUV) were estimated within ROI. Blood-pool activity was used as a background with assessment of SUV maximal and mean levels within the same ROI in the left atrium. Maximal, mean and maximal/mean ratios between SUV mean target and SUV mean background (TBR max, TBR mean and TBR max/mean) were calculated. CT calcium scoring was performed using dedicated software and expressed as Agatston units.

### Follow-Up

During follow-up visit all patients underwent general clinical assessment and echocardiography as the first-line imaging modality in AS. Follow up was performed in 18 ± 6 months after the baseline examination. The analysis of inter-observer reproducibility of peak velocity and mean gradient measurements in patients with AS (based on 20 echocardiographic examinations assessed by 25 different experts) demonstrated better reproducibility of peak aortic velocity (Vmax) compared with mean gradient assessment, suggesting that Vmax should be the preferred indicator to evaluate AS progression (Sacchi et al., 2018). Significant AS progression was defined as deterioration during follow up to the moderate or severe AS in patients with previously mild AS, or identification of severe AS in patients with previously moderate AS, along with increasing of the Vmax more than 0.3 m/s per year or new aortic valve surgery indications.

### Statistical Analysis

Continuous variables were tested for normality by the Shapiro-Wilk test. Results were expressed as mean ± SD, median (percentile 25–75), or percentage as appropriate. Differences between groups were evaluated by a 2-way ANOVA; Wilcoxon rank sum test. Spearman’s rank correlation coefficient was used to assess the relationship between CT, PET and echocardiographic values. The clinical factors associated with fast AS progression were analyzed using the univariate binary logistic regression analysis. Statistically significant variables (*p* < 0.05) were included in multivariate logistic regression analysis. Statistical significance was taken at level *p* < 0.05. The predictive value of model was estimated using ROC analysis. The sensitivity and specificity for the resulting model were also calculated. All analyses were carried out using Statistica 12 and IBM SPSS Statistics 26.0.

## Results

### Clinical Assessment

Patients were divided into two groups according to the valve morphology: BAV and TAV. Aortic valve phenotype was identified by echocardiography and cardiac CT. Patient with TAV were significantly older compared to those with BAVs (Table 1). In TAV and BAV groups male:female ratio was 0.9:1. There were no differences found in systolic and diastolic blood pressure and prevalence of hypertension. Unsurprisingly, incidence of coronary atherosclerosis and diabetes mellitus were higher in TAV patients, as well as statin and antiplatelet therapy use and serum glucose level. Nevertheless, there were not significant differences in lipid profile parameters, that may be related to high dyslipidemia prevalence in both groups.

**TABLE 1:** Clinical characteristics of patients.

	TAV			BAV			p
	Men (*n* = 15)	Women (*n* = 16)	p	Men (*n* = 19)	Women (*n* = 21)	P	
Age, years	63 [57; 65]	64 [62; 67]	0.52	54 [49; 62]	60 [55; 64]	0.33	0.001*
Body mass index, kg/m^2^	28.7 [26.7; 30.6]	31.8 [28.5; 34.2]	0.1	26.7 [25.2; 33.0]	27.2 [25.6; 31.5]	0.7	0.07
Body surface area, m^2^	2.08 [1.94; 2.18]	1.84 [1.74; 2.01]	0.01*	2.14 [2.00; 2.20]	1.82 [1.73; 2.04]	0.01*	0.91
Systolic BP, mm Hg	140 [130; 150]	148 [128; 160]	0.5	130 [120; 140]	130 [125; 150]	0.7	0.72
Diastolic BP, mm Hg	80 [75; 85]	80 [80; 88]	0.13	80 [75; 85]	80 [70; 85]	0.6	0.37
Hypertension, n (%)	14 (93%)	15 (94%)	0,96	15 (79%)	19 (90%)	0.31	0.26
Coronary atherosclerosis, n (%)	12 (80%)	6 (38%)	0,017*	1 (5%)	5 (24%)	0.057	0.0001*
Diabetes mellitus, n (%)	7 (47%)	3 (19%)	0.097	2 (11%)	1 (5%)	0.49	0.007*
Atrial fibrillation, n (%)	4 (27%)	3 (19%)	0.6	2 (11%)	3 (14%)	0.72	0.26
Total cholesterol, mmol/l	4.45 [3.87; 5.42]	5.65 [5.03; 5.91]	0.002*	4.88 [4.03; 5.63]	5.54 [4.78; 6.32]	0.15	0.46
HDL-C, mmol/l	1.12 [1.03; 1.33]	1.49 [1.27; 1.66]	0.002*	1.06 [0.89; 1.41]	1.49 [1.27; 1.66]	0.02*	0.54
LDL-C, mmol/l	2.64 [1.96; 3.22]	3.67 [3.09–3.87]	0.004*	2.91 [2.29; 3.62]	3.55 [2.88–3.96]	0.12	0.53
Glucose, mmol/l	6.01 [5.46; 6.46]	5.70 [5.25; 6.36]	0.49	5.66 [5.14; 5.89]	5.43 [5.21; 5.68]	0.33	**0.034**
eGFR, mL/min/1,73m^2^	76.0 [67.5; 91.2]	73.9 [61.89; 91.78]	0.56	89.1 [75.8; 93.4]	75.84 [65.89; 81.70]	**0.03**	0.44
ACE inhibitors/ARBs, n (%)	13 (87%)	12 (75%)	0.41	14 (74%)	14 (67%)	0.63	0.31
Β-blockers, n (%)	12 (80%)	9 (56%)	0.16	12 (63%)	15 (71%)	0.58	0.98
Warfarin, n (%)	2 (13%)	0	0.13	2 (11%)	1 (5%)	0.49	0.86
DOACs, n (%)				0	2 (10%)	0.17	0.2
Aspirin, n (%)	13 (87%)	9 (56%)	0.06	8 (42%)	8 (38%)	0.8	**0.009**
Statins, n (%)	14 (93%)	11 (69%)	0.08	9 (47%)	9 (43%)	0.77	**0.01**
Echocardiography							
Vmax, m/s	2.8 [2.4; 3.6]	3.0 [2.7; 3.5]	0.5	2.9 [2.6; 3.5]	3.0 [2.5; 3.2]	0.65	0.83
Mean gradient, mm Hg	17.0 [13.2; 29.0]	20.0 [15.5; 27.0]	0.48	22.0 [15.0; 29.0]	22.0 [15.0; 26.0]	0.56	0.92
Index AVA, cm^2^/m^2^	0.59 [0.50–0.88]	0.73 [0.57–0.85]	0.89	0.77 [0.63–0.96]	0.61 [0.54–0.75]	0.12	0.56
LVEF (%)	64 [59; 73]	68 [64; 73]	0.22	64 [60; 66]	65 [63; 67]	0.42	0.09
Index SV (ml/m^2^)	35.4 [31.2; 41.9]	29.0 [25.5; 32.7]	**0.02**	36.9 [27.9; 51.3]	29.7 [23.4; 38.3]	0.06	0.36
AR (moderate, severe), n (%)	1 (7%)	0	0.29	5 (26%)	2 (10%)	0.16	0.06
PET/CT							
Agatston score, AU	1,388 [513; 2,705]	736 [380; 1,153]	0.003*	2,324 [1,038; 3,020]	933 [404; 1,394]	0.004*	0.55
Index Agatston score, AU/m^2^	676 [222; 1,513]	392 [220; 615]	0.017*	1,153 [527; 1,361]	480 [236; 664]	**0.17**	0.42
^18^ F-FDG TBR max	1.18 [1.08; 1.42]	1.10 [1.04; 1.16]	0.014*	1.14 [1.07; 1.24]	1.09 [1.02; 1.16]	0.15	0.39
^18^ F-FDG TBR mean	1.14 [1.03; 1.20]	1.09 [1.01; 1.14]	0.18	1.08 [1.01; 1.16]	1.05 [1.02; 1.11]	0.3	0.49
^18^ F-FDG TBR max/mean	1.54 [1.36; 1.84]	1.39 [1.26; 1.47]	0.031*	1.40 [1.30; 1.55]	1.34 [1.23; 1.49]	0.31	0.4
^18^ F-NaF TBR max	1.65 [1.45; 1.83]	1.44 [1.18; 1.68]	0.033*	1.64 [1.38; 1.88]	1.33 [1.18; 1.82]	0.047*	0.97
^18^ F-NaF TBR mean	1.45 [1.28; 1.65]	1.25 [1.18; 1.41]	0.031*	1.43 [1.32; 1.72]	1.23 [1.15; 1.50]	0.004*	0.83
^18^ F-NaF TBR max/mean	2.03 [1.94; 2.53]	1.89 [1.77; 2.08]	0.033*	2.26 [1.87; 2.67]	1.74 [1.57; 2.64]	**0.059**	0.95

ACE inhibitors, angiotensin-converting-enzyme inhibitors; AR, aortic regurgitation; ARBs, angiotensin receptor blockers; BAV, bicuspid aortic valve; BP, blood pressure; DOACs, direct oral anticoagulants; eGFR - estimated glomerular filtration rate using MDRD formula; HDL-C, high-density lipoprotein cholesterol; iAVA, indexed aortic valve area; LDL-C, low-density lipoprotein cholesterol; LVEF, left ventricular ejection fraction; SV, stroke volume; TAV, tricuspid aortic valve; TBR max, maximal tissue to background ratio; TBR mean, mean tissue to background ratio; TBR max/mean, maximal tissue to mean background ratio; V max, peak aortic jet velocity.

Statistically significant values with *p* less than 0.05 are highlighted.

**FIGURE 1:**
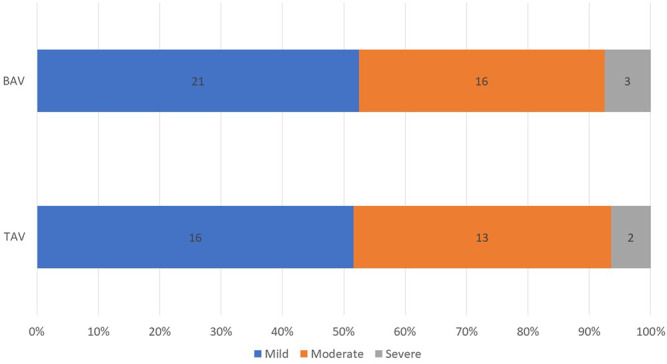
Distribution of AS severity by groups.

In order to examine whether there was a difference in obtained variables according to age analysis of variance for categorical data and analysis of covariance for quantitative characteristics were performed to compare groups regarding to age. After adjusting for age, serum glucose level in both groups did not differ. When comparing TAV and BAV patients while considering age, results didn’t show statistically significant difference for hypertension, coronary atherosclerosis and diabetes mellitus. TAV and BAV patients were matched for the major echocardiography AS severity parameters: Vmax, mean gradient and iAVA. There were no differences revealed by groups in EF, indexed SV and moderate-to-severe AR incidence. Distribution according to the AS severity was comparable in both groups (Figure 1).

**FIGURE 2:**
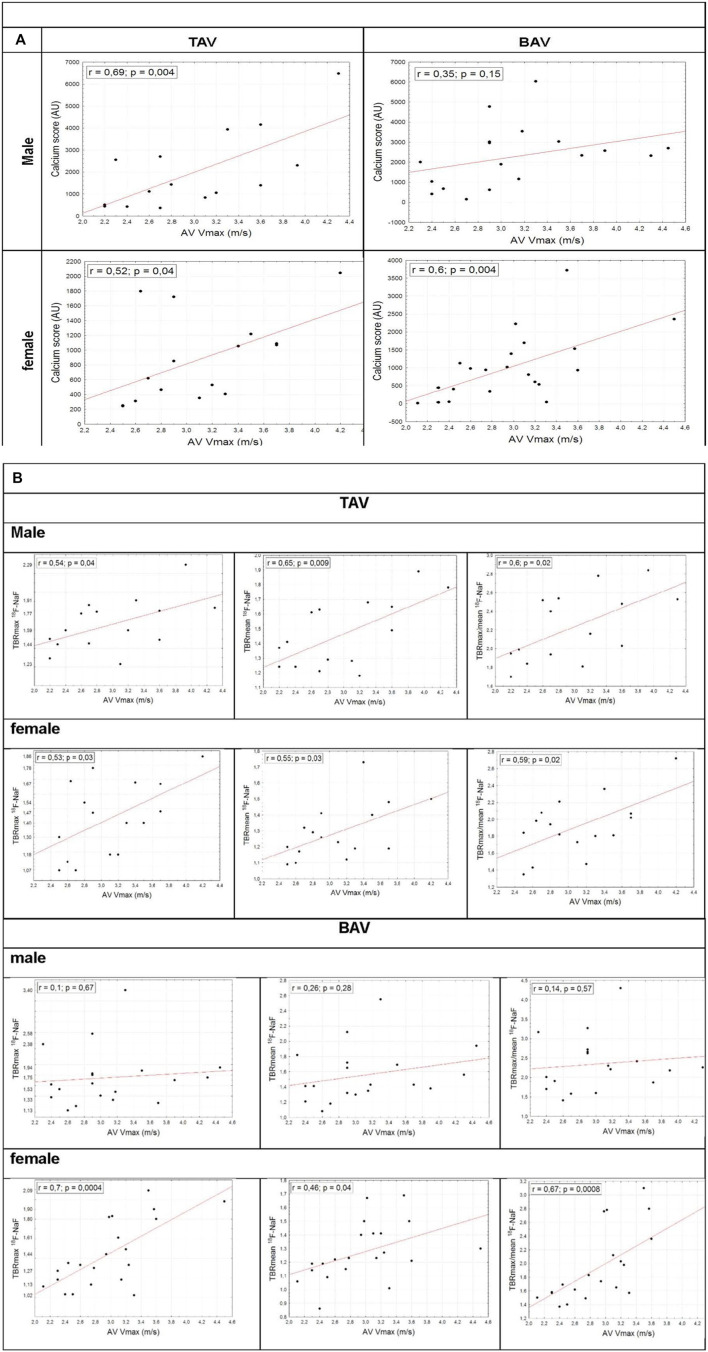
Correlations between Vmax and Agatston score **(A)**, V max and ^18^F-NaF TBR max, ^18^F-NaF TBR mean, ^18^F-NaF TBR max/mean in BAV and TAV groups **(B)**.

Males and females in both groups were matched for age, body mass index, systolic and diastolic blood pressure, prevalence of hypertension and diabetes mellitus. Body surface area and creatinine level were predictably higher in men. Glomerular filtration rate was lower in women in BAV group, at the same time both male and female patients did not suffer significant chronic renal disease. In TAV group worse lipid profile in women may be explained by coronary atherosclerosis frequency, and thus intensive statin therapy in men. Both groups were matched in medical therapy. Hemodynamic severity of AS determined by Vmax, mean transvalvular gradient, iAVA was comparable in men and women in TAV and BAV groups. Both groups did not differ in valvular calcification value (Agatston score) and disease activity (^18^F-NaF TBR max, ^18^F-NaF TBR mean, max/mean TBR). The degree of valvular inflammation estimated by ^18^F-FDG TBR max, TBR mean and TBR max/mean also did not differ in TAV and BAV patients. Additionally inflammatory tracer activity did not differ in groups despite ACE inhibitors/ARBs or statins treatment. Predictably, in both groups aortic valve Agatston Score was higher in men. Furthermore, PET/CT demonstrated more advanced calcification in male patients. In TAV patients ^18^F-FDG accumulation was higher in men, that possibly related to greater prevalence of the coronary atherosclerosis and systemic chronic inflammation in this subgroup. Agatston score and ^18^F-NaF TBR max, ^18^F-NaF TBR mean, ^18^F-NaF TBR max/mean values correlated with AS severity estimated by Vmax in men and women with TAV. In BAV group this association was true only in female patients (Figure 2).

### Follow up

All 71 patients underwent clinical and echocardiographic evaluation during follow-up visits. The mean time interval between the two echocardiograms was 16.8 ± 4.2 months. Doppler transthoracic echocardiography was performed to all patients.

Following the results, the rate of AS progression varied highly between patients. During this time period the mean systolic gradient and Vmax value increased from 22.3 to 25.8 mm Hg and from three to 3.2 m/s respectively, while iAVA parameter was stable (0.73 cm^2^/m^2^). The annual growth rate of Vmax value was 0.11 ± 0.27 m/s per year.

**FIGURE 3:**
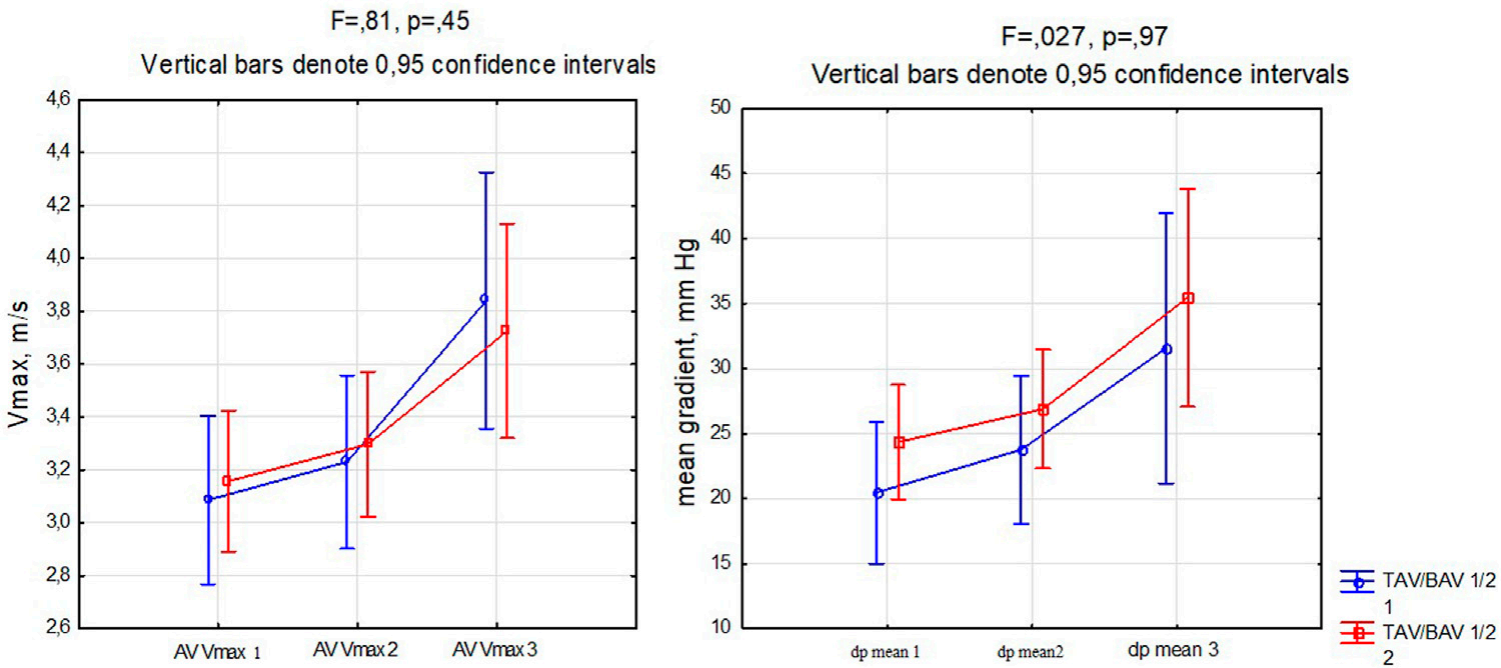
V max and mean gradient dynamics by groups during follow up.

There was also third follow up visit in 34 patients. Mean time period was 46 months (from 22 to 74 months). It was demonstrated that progression rates measured by Vmax and mean gradient did not differ between groups (Figure 3).

Significant AS progression was defined as deterioration during follow up to the moderate or severe AS in patients with previously mild AS, or identification of severe AS in patients with previously moderate AS, along with increasing of the Vmax more than 0.3 m/s per year or new aortic valve surgery indications. Among all participants 29 (41%) patients fulfilled such criteria.

The clinical predictors of AS progression were analyzed using the binary logistic regression. Based on the univariate binary logistic regression analysis data, it was demonstrated that baseline AS severity, V max, mean gradient, iAVA, aortic valve Agatston Score, ^18^F-NaF TBR, coronary atherosclerosis and use of statins and antiplatelet agents contributed to rapid progression of AS (Table 2). Notable, that in BAV patients iAVA was not associated with AS progression that possible related to technical complexity of AVA quantification due to not truly circular left ventricular outflow tract.

**TABLE 2:** Results of univariate binary logistic regression analysis to investigate predictors of AS progression.

	As (*n* = 71)			TAV (*n* = 31)			BAV (*n* = 40)		
	OR	95% CI	p	OR	95% CI	p	OR	95% CI	P
Age	1.06	0.99–1.14	0.07	1.001	0.88–1.14	0.99	1.08	0.99–1.18	0.09
Male	0.61	0.24–1.58	0.31	0.68	1.66–2.8	0.59	0.55	0.14–2.05	0.37
Smoking	1.3	0.39–4.34	0.67	2.55	0.69–16.55	0.33	0.7	0.12–4.18	0.7
Obesity	1.15	0.43–3.01	0.78	1.47	0.36–6.05	0.59	0.76	0.18–3.1	0.7
Hypertension	1.17	0.26–5.33	0.84	0.93	0.53–16.39	0.96	1.09	0.17–6.85	0.92
TAV	0.57	0.22–1.5	0.26						
Diabetes mellitus	1.91	0.57–6.41	0.3	2	0.43–9.53	0.38	0.92	0.76–11.17	0.95
Coronary atherosclerosis	**2.83**	**1.03–7.73**	**0.043**	2.76	0.58–12.98	0.2	2.2	0.46–10.62	0.33
Cholesterol	0.92	0.68–1.25	0.61	0.93	0.65–1.33	0.7	0.89	0.53–1.51	0.68
LDL-C	0.81	0.55–1.2	0.3	0.88	0.59–1.32	0.54	0.67	0.34–1.32	0.25
HDL-C	0.99	0.24–4.14	0.99	0.52	0.04–6.89	0.62	1.31	0.23–7.53	0.76
CRP	0.99	0.9–1.1	0.91	1.08	0.91–1.28	0.4	0.89	0.67–1.17	0.38
GFR	1.01	0.98–1.05	0.49	1.01	0.97–1.06	0.64	1.02	0.97–1.08	0.51
β-blockers	1.93	0.67–5.56	0.22	1.65	0.36–7.6	0.52	2.29	0.51–10.28	0.28
ACE inhibitors/ARBs	3.13	0.91–10.75	0.07	2.17	0.33–14.06	0.42	3.75	0.69–20.38	0.13
Calcium channel blockers	0.81	0.3–2.22	0.68	0.64	0.15–2.77	0.55	0.9	0.22–3.75	0.89
Statins	**13.5**	**2.84–64.15**	**0.001**			0.99	**9.6**	**1.77–52.17**	**0.009**
Aspirin	**2.96**	**1.09–8.02**	**0.03**	1.25	0.26–5.94	0.78	**4.89**	**1.21–19.71**	**0.02**
Warfarin	2.31	0.36–14.77	0.38			0.99	0.92	0.08–11.17	0.95
Vmax	**8.44**	**2.65–26.89**	**0.000**	**19.81**	**2.4–163.18**	**0.006**	**5.2**	**1.31–20.61**	**0.02**
mean gradient	**1.14**	**1.06–1.23**	**0.000**	**1.24**	**1.06–1.45**	**0.006**	**1.12**	**1.02–1.21**	**0.02**
iAVA	**0.03**	**0.002–0.56**	**0.02**	**0.003**	**0.00–0.62**	**0.03**	0.11	0.004–2.12	0.16
Severity	**3.7**	**1.55–8.82**	**0.003**	**4.35**	**1.09–17.28**	**0.04**	**3.46**	**1.1–10.84**	**0.03**
index SV	0.97	0.92–1.01	0.16	0.97	0.88–1.08	0.6	0.97	0.92–1.03	0.28
IMMLV	1.01	0.99–1.02	0.17	1.02	0.99–1.05	0.21	1.01	0.99–1.02	0.35
EF	0.98	0.91–1.05	0.49	0.94	0.86–1.04	0.24	1.004	0.89–1.13	0.95
Calcium Score	**1.001**	**1–1.001**	**0.014**	**1.002**	**1.001–1.004**	**0.01**	**1**	**1–1.001**	**0.34**
Calcium Score/m2	**1.001**	**1–1.002**	**0.009**	**1.004**	**1.001–1.007**	**0.01**	**1.001**	**1–1.002**	**0.28**
^18^F-NaF TBR max	**7.06**	**1.55–32.27**	**0.01**	**3652.59**	**14.3–932,992.9**	**0.004**	2.45	0.59–10.16	0.22
^18^F-NaF TBR mean	**27.47**	**2.96–254.47**	**0.004**	**38,979.76**	**26.38–576,021.09**	**0.01**	6.2	0.67–57.09	0.11
^18^F-NaF TBR max/mean	**4.88**	**1.66–14.36**	**0.004**	**681.93**	**5.19–89,651.37**	**0.009**	2.48	0.83–7.38	0.1
^18^F-FDG TBR max	17.72	0.63–497.02	0.09	29.84	0.24–3764.89	0.17	7.48	0.05–1030.57	0.42
^18^F-FDG TBR mean	6.78	0.12–373.06	0.35	29.76	0.1–8701.57	0.24	0.16	0.00–332	0.64
^18^F-FDG TBR max/mean	6.1	0.64–58.57	0.12	21.12	0.55–806.95	0.1	1.72	0.08–36.22	0.73

ACE inhibitors, angiotensin-converting-enzyme inhibitors; ARBs, angiotensin receptor blockers; BAV, bicuspid aortic valve; BP, blood pressure; CRP, C-reactive protein, DOACs, direct oral anticoagulants; EF, ejection fraction; eGFR, estimated glomerular filtration rate using MDRD formula; HDL-C, high-density lipoprotein cholesterol; iAVA – indexed aortic valve area; IMMLV, indexed myocardial mass of left ventricle; LDL-C, low-density lipoprotein cholesterol; LVEF, left ventricular ejection fraction; SV, stroke volume; TAV, tricuspid aortic valve; TBR max, maximal tissue to background ratio; TBR mean, mean tissue to background ratio; TBR max/mean, maximal tissue to mean background ratio; V max, peak aortic jet velocity.

Statistically significant values with *p* less than 0.05 are highlighted.

**FIGURE 4:**
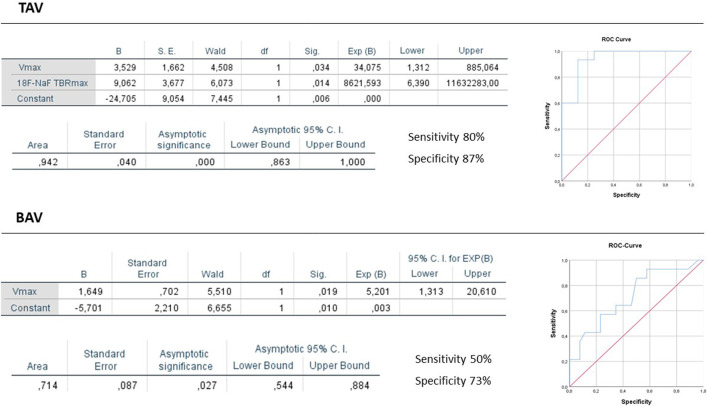
Results of the ROC analysis in all patient and TAV and BAV groups.

**FIGURE 5:**
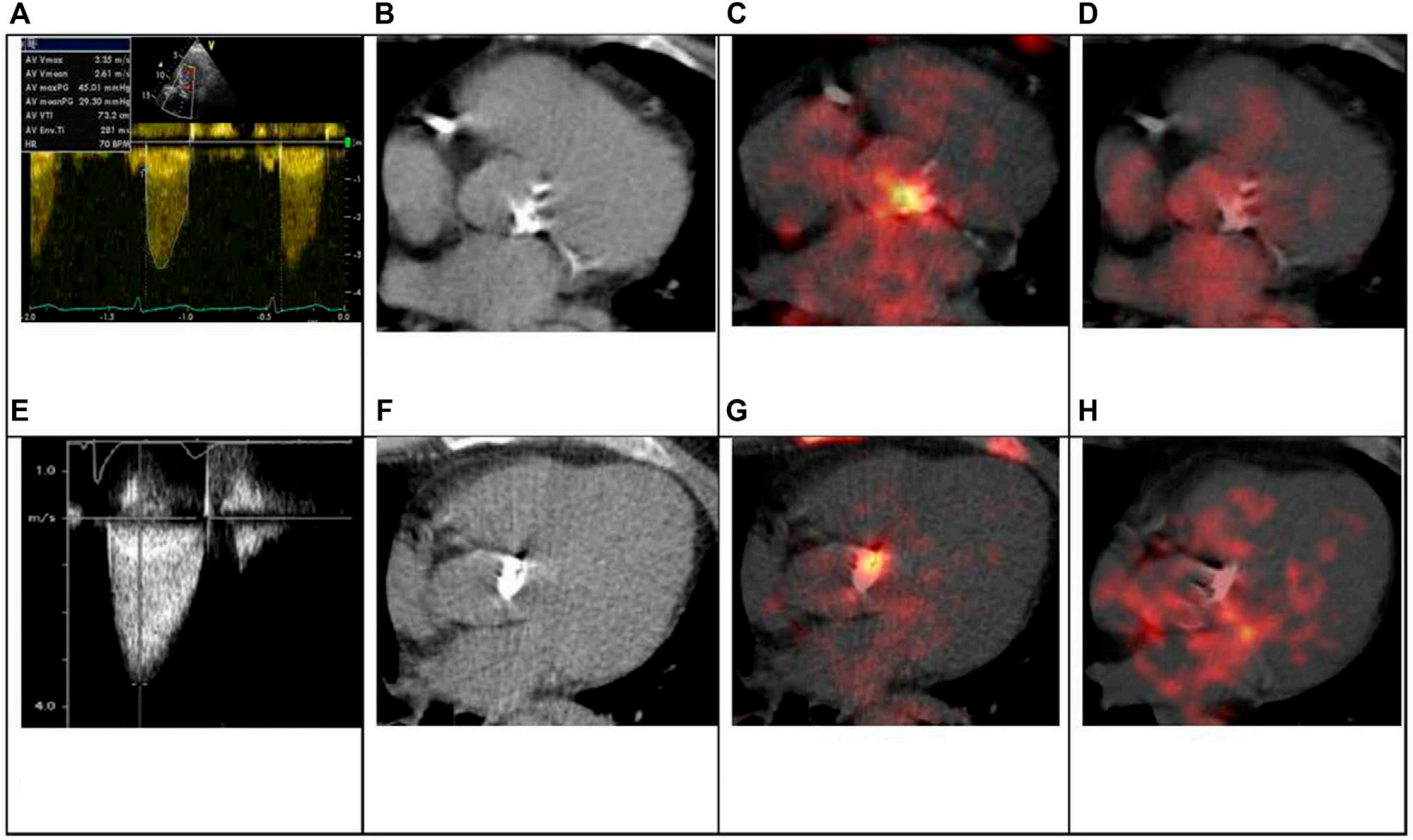
TAV and BAV patient with moderate AS. 64-years old asymptomatic patient with TAV. Echo parameters: Vmax 3.35 m/s, mean gradient 29 mmHg **(A)**. CT revealed and severe aortic valve calcification: Agatston Score 3139 AU **(B)**. ^18^F-NaF PET/CT demonstrated marked accumulation of the radiotracer: ^18^F-NaF TBR max 1.91 **(C)**. ^18^F-FDG valve accumulation was not significant, ^18^F-FDG TBR max 1.05 **(D)**. 2 years hence symptoms manifested, Vmax increased to 4.2 m/s and aortic valve replacement was performed. 51-years old patient with BAV. Echo parameters: Vmax 3.4 m/s, mean gradient 30 mmHg **(E)**. CT revealed and severe aortic valve calcification: Agatston Score 4769 AU **(F)**. ^18^F-NaF PET/CT demonstrated significant accumulation of the radiotracer: ^18^F-NaF TBR max 2.58 **(G)**. ^18^F-FDG valve uptake was not increased, ^18^F-FDG TBR max 1.17 **(H)**. There was no clinical or hemodynamic progression registered during 4-years follow up.

The multivariate binary logistic regression analysis of these predictors was performed to evaluate independent predictors of AS progression. In TAV group close correlation between Agatston Score and ^18^F-NaF TBRmax was observed (r = 0.78; *p* < 0.001). Due to multicollinearity of these parameters, we included in the multivariate regression analysis ^18^F-NaF TBRmax. The most important positive predictors of AS progression in TAV obtained by multivariate logistic regression analysis were Vmax and ^18^F-NaF TBRmax. Whereas in BAV the highest predictive value showed model included Vmax (Figure 4). All presented indicators showed good predictive value. Figure 5 demonstrates clinical cases of TAV and BAV patient with moderate AS and different progression rates of valvular heart disease.

## Discussion

It was demonstrated that baseline AS severity (measured by V max) correlated with high risk of disease progression, that was in accordance with previous studies (Bohbot et al., 2017; Yang et al., 2021). The effect of aortic valve phenotype on the AS progression remains uncertain. Most of the earlier studies that analyzed the factors associated with AS progression did not evaluate the role of valve phenotype. Most commonly, TAV and BAV patients were analyzed together, with a large predominance of TAV patients and/or comorbidities that differed greatly between men and women. The few studies that assessed the impact of valve phenotype on AS progression demonstrated no association between BAV and progression rate (Chan et al., 2010; Nguyen et al., 2015). In our cohort, men and women in both groups were well matched for all clinical data and hemodynamic severity of AS. In TAV and BAV patients progression rates were similar.

Over the last decade it has become possible to evaluate AS pathogenesis *in vivo*. The degree of valve inflammation, measured by ^18^F-FDG accumulation, is considered to be most crucial at the early stages of the AS, while high ^18^F-NaF accumulation, representing microcalcification activity, is known to be a strong predictor of AS progression (Dweck et al., 2012; Dweck et al., 2014). Nevertheless, inhibitors of calcification pathway (denosumab and alendronic acid) were found to be ineffective in the context of inhibition of AS progression measured by Vmax, Agatston Score and ^18^F-NaF. However, this study did not take into account valve phenotype and gender differences in AS calcification (Pawade et al., 2021).

We estimated the role of the main pathological processes—inflammation and calcification in AS progression with consideration to the traditional cardiovascular risk factors in TAV and BAV patients. Our study demonstrated comparable valve calcification degree and microcalcification activity in patients with different aortic valve morphology. Moreover, in TAV and BAV groups the rate of ^18^F-FDG accumulation did not correlate with AS severity, while ^18^F-NaF accumulation was associated with both echocardiography parameters of AS severity and aortic valve Agatston index by CT. However, males with BAV did not demonstrate correlation between AS severity, aortic valve calcification degree and disease activity. Multivariate analysis revealed that rapid AS progression in TAV patients was determined by baseline AS severity, and calcification activity by of ^18^F-NaF PET/CT. While in BAV patients baseline aortic valve calcification did not influence on AS prognosis whereas Vmax value along with age were the main predictors of AS progression. Biological background for this phenomenon is still unclear but might be due to different significance of provocative aortic valve calcification factors in women and men, such as vitamin D receptors and growth factors (Aggarwal et al., 2013). Taking this into consideration, therapy, targeted on inhibition of calcification mechanisms, seems to be more beneficial for patients with TAV.

To date, there are no effective medical therapies to delay AS progression and the only available treatment option for symptomatic severe AS remains surgical or transcatheter aortic valve replacement with a mechanical or bioprosthetic valve. However, these two techniques may be associated with significant complications: the implantation of a mechanical valve increases the risk of thrombosis and requires a life-long anticoagulation therapy. On the other hand, the bioprosthetic valves are prone to structural deterioration, resulting in limited long-term durability that can lead to reoperation in less than 15 years (Zhao et al., 2016). Effective medical treatment is required to reverse the progression of AS and to reduce the need for aortic valve replacement that would contribute to better clinical outcome in such patients. Novel drug therapies should target valve-specific and sex-related signaling pathways to be more effective.

Our results should be interpreted in the context of certain limitations.

The study population is not large enough to exclude the contribution of other risk factors to AS progression. While the study points to several new risk factors, the mechanisms by which they operate are not clear. It is important that groups were not matching by age. Despite we took into account age as a cofactor, its influence cannot be completely ruled out. The progression of AS was presumed to be linear based on follow up echocardiography scans over an average of 18 ± 6 months. However, it is possible that AS progression was not linear and the correlation between AS progression and aortic valve calcification was variable with different intervals.

In conclusion, ^18^F-NaF PET/CT may be considered as the valuable predictor for hemodynamic progression of calcific AS in case of TAV. ^18^F-FDG PET/CT does not play a significant role to predict the development of severe aortic stenosis.

## Data Availability

The original contributions presented in the study are included in the article/supplementary material, further inquiries can be directed to the corresponding author.
